# Vascular embolization as an adjunct to surgical removal of percutaneously inserted foreign bodies in the pharynx to avoid the risk of massive bleeding: a case report

**DOI:** 10.3389/fsurg.2026.1774286

**Published:** 2026-05-21

**Authors:** Huaizhuang Cai, Jingqin Cao, Qian Wang

**Affiliations:** Department of Interventional Radiology, Jining No. 1 People's Hospital, Jining, Shandong, China

**Keywords:** case report, embolization, foreign body, hemorrhage, parapharyngeal space, surgery

## Abstract

**Rationale:**

Percutaneous penetrating foreign bodies in the parapharyngeal space complicated by arterial injury are rare but pose a high risk of life-threatening hemorrhage, and optimal management strategies remain unclear.

**Patient concerns:**

A 32-year-old man presented with a 4-day history of left infra-auricular swelling and a 1-day history of recurrent hematemesis after accidental penetration by a bamboo chopstick. Computed tomography showed a linear foreign body in the left parapharyngeal space, accompanied by surrounding tissue swelling and pharyngeal narrowing.

**Diagnosis:**

The patient was diagnosed with a percutaneous penetrating foreign body in the left parapharyngeal–lateral skull base region, with suspected vascular injury.

**Interventions:**

The patient underwent preoperative endovascular embolization of the left maxillary artery, followed by surgical removal of the foreign body and debridement under general anesthesia.

**Outcomes:**

Angiography confirmed active bleeding from the maxillary artery. After embolization, the foreign body was successfully removed with minimal intraoperative blood loss (approximately 5 mL). The patient recovered without complications.

**Lessons:**

In selected cases of penetrating parapharyngeal foreign bodies with arterial injury, preoperative embolization may reduce intraoperative bleeding risk and improve surgical safety.

## 1 Introduction

Pharyngeal foreign bodies are a common otolaryngologic emergency, typically entering through the oral cavity (1, 2). In contrast, percutaneous penetrating pharyngeal foreign bodies complicated by major artery injury are relatively rare but carry a high risk of fatal bleeding during treatment. The parapharyngeal space is a deep potential space in the head and neck, located adjacent to critical structures, including the internal and external carotid artery branches and cranial nerves. Although foreign bodies in this region are uncommon, they pose a significant clinical risk and, if untreated, may lead to deep infection, vascular injury, severe bleeding, or other life-threatening complications (3, 4).

Traditional surgical removal of parapharyngeal foreign bodies is challenging and hazardous because of the deep anatomical location, limited surgical exposure, and risk of massive bleeding from vascular injury. In recent years, endovascular techniques, particularly transcatheter arterial embolization, have been increasingly used to manage head and neck vascular injuries and acute hemorrhage. These techniques enable precise identification and occlusion of injured vessels, thereby reducing intraoperative bleeding and improving surgical safety (5). Despite these advantages, the use of preoperative embolization for penetrating parapharyngeal foreign bodies has been rarely reported, and no consensus exists on optimal management.

In this context, we report the successful removal of a long bamboo chopstick from the parapharyngeal space using a combined approach of vascular embolization and surgery and discuss the clinical value of this approach to inform the management of similar cases.

## 2 Case presentation

A 32-year-old man was admitted with a 4-day history of left infra-auricular swelling and a 1-day history of recurrent hematemesis. Four days earlier, he had fallen after alcohol consumption and sustained a puncture injury to the left earlobe from a bamboo chopstick. He reported left ear and throat pain without significant bleeding from the wound or ear canal, hemoptysis, or hematemesis, and did not receive any treatment. One day before admission, he developed progressively worsening infra-auricular swelling and throat pain, followed by sudden hematemesis of approximately 100 mL of fresh blood. Although the bleeding stopped spontaneously, it recurred and was accompanied by trismus and difficulty with oral intake. On examination, his temperature was 37.8℃, heart rate was 92 bpm, respiratory rate was 22 breaths/min, blood pressure was 125/80 mmHg, and oxygen saturation was 98%. Breath sounds were clear bilaterally, with no rales or chest friction rubs.

### 2.1 Diagnostic assessment

**Figure 1:**
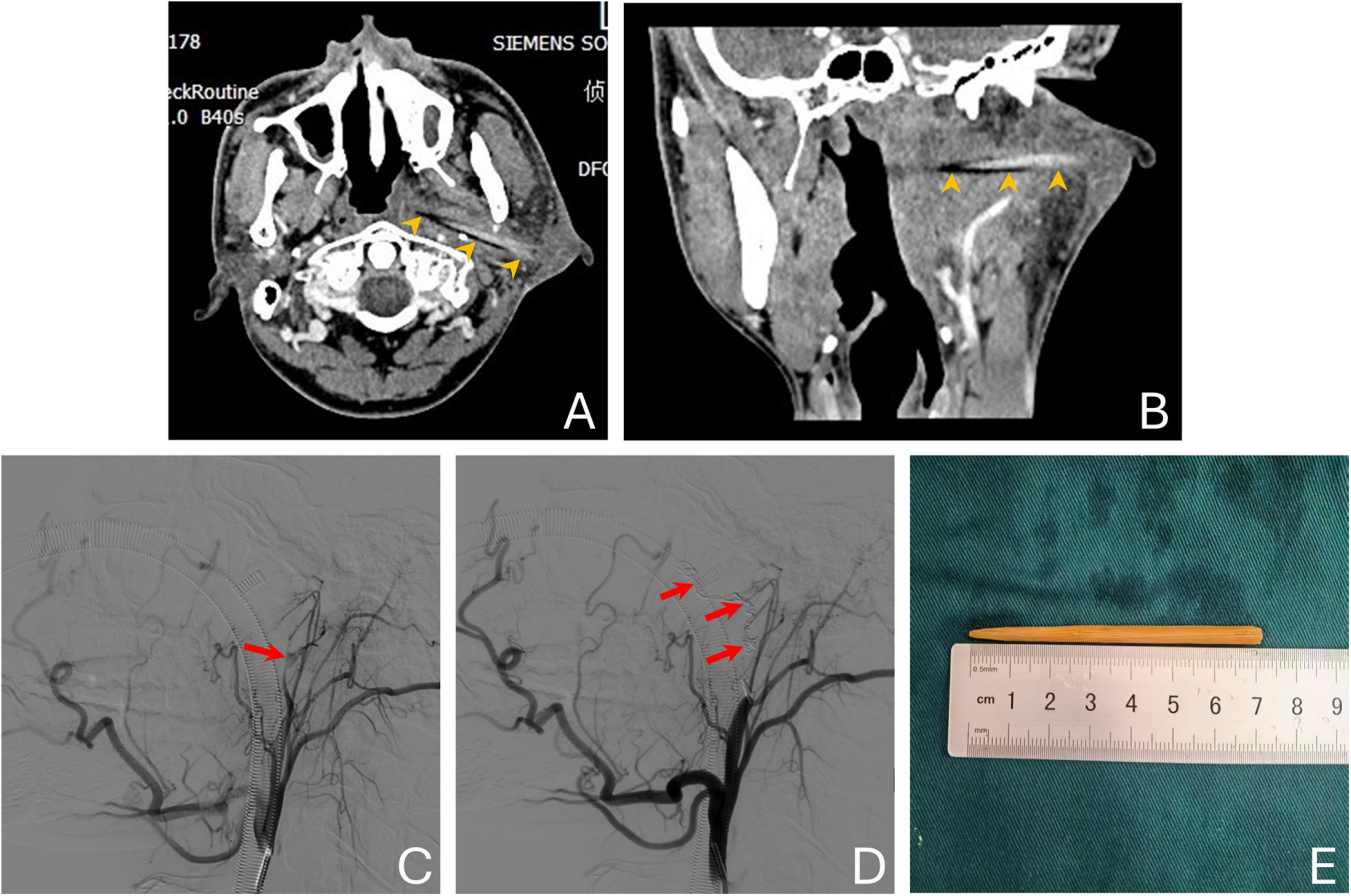
**(A,B)** Oropharynx CT scan showing significant swelling extending from below the left auricle to the left parapharyngeal space, with a strip-shaped high-density shadow inside (orange arrowhead) and associated pharyngeal stenosis. **(C,D)** Intraoperative facial angiography revealing local extravasation of the contrast agent (red arrow) from the maxillary artery **(C)**, and several coils (red arrow) were used to perform maxillary artery embolization **(D)**. **(E)** Image of the foreign body causing damage to the maxillary artery.

Oropharyngeal computed tomography (CT) scanning showed significant swelling extending from the region below the left auricle to the left parapharyngeal space, along with a linear high-density foreign body and associated pharyngeal stenosis (Figures 1A,B). The preliminary diagnosis was a foreign body in the left parapharyngeal–lateral skull base region. The object had entered the subcutaneous tissue below the left earlobe, traversed the parotid gland, and extended anteromedially into the left parapharyngeal space. It lay close to the left maxillary artery anteriorly, abutted the left styloid process posteriorly, approached the left cranial base superiorly, and was located near the left medial pterygoid muscle inferiorly.

Under general anesthesia, the right femoral artery was accessed using the Seldinger technique, and a 5F VER catheter was introduced. The left common carotid artery was selectively cannulated for angiography, which showed contrast extravasation from the proximal left maxillary artery (Figure 1B). A microcatheter was then advanced through the guide catheter to superselect the left maxillary artery; after confirming its position, embolization was performed under fluoroscopic guidance using two Boston Scientific coils (models: Interlock-18-4-10 and Interlock-18-4-6) and two Cook Medical coils (model: MWCE-18S-3.0-3-HILAL) delivered through the microcatheter. Subsequent angiography confirmed complete hemostasis (Figure 1C).

The foreign body located in the parapharyngeal space and lateral skull base was then removed, followed by debridement and suturing of the infected maxillofacial soft tissues. A curvilinear incision was made from the skin laceration site anteroinferior to the left earlobe. Upon incision, dark red hemorrhagic and purulent secretions were observed within the subcutaneous layer and were thoroughly cleared; the underlying tissues were disrupted, and the boundaries between the parotid capsule and surrounding tissues were indistinct. The foreign body was palpated approximately 1.0 cm beneath the skin surface. The tissues surrounding the foreign body were carefully dissected to expose approximately 1.5 cm of one end of the object. The foreign body was then gently loosened, firmly grasped with a vascular clamp, and extracted opposite to its insertion path under imaging guidance (the object was identified as a bamboo chopstick measuring approximately 7.0 cm). The extracted fragment was compared with the remaining portion of the chopstick provided by the relatives of the patient, and the fractured ends matched perfectly. Following removal of the foreign body, no active bleeding was observed within the surgical cavity; the incision was closed in layers, and a drainage tube was inserted.

Postoperatively, the patient was treated with cefoperazone and sulbactam sodium for infection control, mannitol for swelling reduction, and hemostatic agents for bleeding prevention. Dressings were changed regularly, and the drainage tube was removed 48 h postoperatively. The patient reported no postoperative complications, including fever, bleeding, or dyspnea. Mouth opening gradually recovered, and by 18 days postoperatively, the swelling had completely resolved, mouth opening had returned to normal, and the patient was discharged with full recovery.

### 2.2 Follow-up

At the 3-month follow-up, the patient reported no discomfort, including sore throat, tinnitus, or facial numbness. Follow-up oropharyngeal CT scanning revealed clear parapharyngeal space structures without any abnormal lesions.

## 3 Discussion

Percutaneous penetrating foreign bodies in the parapharyngeal space are rare but potentially life-threatening because of the dense concentration of critical neurovascular structures, including the internal and external carotid arteries, jugular vein, and cranial nerves. Vascular injury in this region significantly increases the risk of massive hemorrhage and perioperative complications, making optimal management both challenging and essential (6).

Studies have reported that management strategies for penetrating foreign bodies in the deep neck spaces primarily include surgical removal (7), image-guided extraction (8), or conservative observation in selected asymptomatic cases (9). However, when vascular injury is suspected or confirmed, the risk of uncontrolled bleeding during surgical exploration becomes a major concern. Previous studies have highlighted the importance of preoperative vascular assessment using contrast-enhanced CT, CT angiography, or digital subtraction angiography to identify arterial involvement and guide intervention planning (10, 11). CT angiography is widely used as a non-invasive modality for evaluating vascular injury, whereas digital subtraction angiography enables precise diagnosis and immediate therapeutic intervention in cases of suspected active bleeding.

In this context, the combination of preoperative embolization and surgical removal, as demonstrated in this case, represents a rational and increasingly recognized strategy. Preoperative embolization effectively occludes injured or high-risk vessels, thereby significantly reducing intraoperative hemorrhage and improving surgical safety. Similar approaches have been reported in the management of traumatic vascular injuries and hypervascular head and neck tumors, in which embolization serves as an adjunct to facilitate safer resection (12–14). Our case further supports the feasibility of this strategy for penetrating foreign bodies associated with arterial involvement.

Compared with immediate surgery alone, the addition of embolization offers several advantages. First, it enables precise localization and characterization of vascular injury. Second, it reduces intraoperative blood loss and operative time. Third, it may decrease the risk of emergency vascular repair or catastrophic bleeding. However, embolization is not without risks. Potential complications include non-target embolization, ischemic injury to adjacent tissues, cranial nerve deficits, and, rarely, stroke (14, 15). Therefore, careful patient selection, thorough angiographic evaluation, and experienced interventional techniques are essential to minimize these risks.

Alternative management strategies should also be considered. In the absence of vascular involvement, minimally invasive image-guided removal may suffice, avoiding the risks associated with surgery and embolization. Conversely, unstable patients with active hemorrhage may require immediate surgical or endovascular intervention. Endovascular techniques, including stent-graft placement, have also been used in the management of certain vascular injuries and may serve as an alternative to embolization in selected cases (5, 16). Therefore, treatment should be individualized based on the location of the foreign body, the extent of vascular injury, and the overall clinical condition of the patient.

Despite the favorable outcome observed in this case, some limitations should be acknowledged. First, this single case report limits the generalizability of the findings. Second, the relatively short follow-up period leaves long-term outcomes, including delayed vascular complications, uncertain. Third, the lack of comparative data prevents definitive conclusions regarding the superiority of the combined approach over other management strategies.

In conclusion, this case highlights the importance of comprehensive preoperative evaluation and a multidisciplinary approach in the management of penetrating parapharyngeal foreign bodies associated with vascular injury. In selected patients, the combination of preoperative embolization and surgical removal appears safe and effective that may improve hemorrhage control and surgical outcomes. Therefore, further studies involving larger case series are needed to validate this approach and establish standardized treatment guidelines.

## Data Availability

The original contributions presented in the study are included in the article/Supplementary Material, further inquiries can be directed to the corresponding author.
